# Successful Treatment of Oxaliplatin‐Induced Immune Thrombocytopenia in a Schizophrenic Patient With Contraindication to Steroids Using Cyclosporine Plus Eltrombopag: A Case Report

**DOI:** 10.1155/carm/9589387

**Published:** 2026-01-28

**Authors:** Jian Zheng, Nan Zhou, Dexiang Pang, Shaoling Zhou, Huajun Zheng

**Affiliations:** ^1^ Oncology Department, The Second Affiliated Hospital of Zhejiang Chinese Medicine University, Hangzhou, Zhejiang Province, China; ^2^ Oncology Department, Hangzhou Linping District Traditional Chinese Hospital, Hangzhou, Zhejiang Province, China; ^3^ Gastroenterology Department, The Second Affiliated Hospital of Zhejiang Chinese Medicine University, Hangzhou, Zhejiang Province, China

**Keywords:** cyclosporine, drug-induced thrombocytopenia, eltrombopag, immune thrombocytopenia, oxaliplatin

## Abstract

**Background:**

Oxaliplatin is widely used in the treatment of gastrointestinal tumors but is associated with rare adverse effects, including immune thrombocytopenia. The incidence of oxaliplatin‐associated immune thrombocytopenia (OIIT) may be frequently overlooked due to its rarity, and in some cases, it can be difficult to treat. We present our successful experience with the use of combination therapy consisting of cyclosporine and eltrombopag in one patient, who was not suitable for steroid medication due to schizophrenia and had failed to respond when treated with eltrombopag alone.

**Case Presentation:**

A 57‐year‐old Chinese woman with colon cancer suffered from schizophrenia, whereas thrombocytopenia had never been demonstrated. She experienced persistent thrombocytopenia and recurrent bleeding at mucocutaneous sites after oxaliplatin‐containing chemotherapy. Then, oxaliplatin‐induced immune thrombocytopenia was diagnosed after almost all necessary examinations. The medical history limits the use of long‐term hormone therapy. Her condition remained uncontrolled following a range of treatments regimen that involved eltrombopag alone. However, after initiating a combination of agents such as cyclosporine and eltrombopag, her platelet count normalized within 6 months, and she experienced no relapses during a 2‐year follow‐up period.

**Conclusion:**

Our results suggest that combination therapy with cyclosporine and eltrombopag exhibits better response rates in settings of the existence of contraindications to other therapies and less efficacy of eltrombopag alone. This knowledge will be helpful for other clinicians to choose an appropriate treatment in such difficult circumstances.

## 1. Introduction

Oxaliplatin, a platinum‐coordinated​ chemotherapeutic agent, is pivotal in the management of malignant tumors of the digestive tract. Myelosuppression represents the main predominant mechanism in thrombocytopenia induced by oxaliplatin. Oxaliplatin‐associated immune thrombocytopenia (OIIT) is an infrequent event and can be easily underreported. The exact pathogenesis may be associated with autoimmune disturbance and production of autoantibodies against the platelets [1]. A minority of the patients develop a chronic course which may protracted, some assuming refractory states. In general, the guiding therapeutic strategy comes from the recommendations on immune thrombocytopenia (ITP). Corticosteroids and immunoglobulins provide quick symptomatic improvement but are not advised for continuous treatment, especially in patients with accompanying psychic disorders. Other treatment options considered effective for the induction of long‐term disease control and remission include splenectomy, thrombopoietin receptor agonists, and immunosuppressive therapy.

Herein, we would like to present our effective clinical experience using a combination therapy that includes cyclosporine and eltrombopag for a patient with oxaliplatin‐induced ITP. This patient was considered unsuitable for steroid treatment because of her schizophrenia diagnosis and showed an insufficient response to eltrombopag monotherapy.

## 2. Case Report

The case analyzed here involves a 57‐year‐old female with a history of schizophrenia and appendectomy. She has no history of tobacco use, alcohol consumption, or drug/food allergies. The prior physical examination did not indicate any hematological disorders, and there is no significant family history to note. The patient was diagnosed with Stage IIIB rectal cancer, which was attributed to unspecified hematochezia, and subsequently underwent laparoscopic radical resection of the rectal cancer on August 26, 2021. Following the surgery, from September 16, 2021, to January 25, 2022, she received six rounds of adjuvant chemotherapy using the oxaliplatin and capecitabine regimen. Unfortunately, she developed Grade 4 thrombocytopenia as a side effect of the treatment, with a lowest recorded platelet count of 5 × 10^9^/L, and also experienced dermal mucosal bleeding during this period. On February 26, a bone marrow aspiration analysis revealed a significant presence of myeloblasts and a notable number of megakaryocytes (29 megakaryocytes), with no change in the immunophenotypic profile. Also, bone marrow biopsy pathology showed that hematopoietic tissue proliferation is very active. Despite implementing various measures—including discontinuing chemotherapeutic agents, administering drugs that induce thrombocytosis, providing platelet transfusions, and adjusting psychoactive drug olanzapine to risperidone and oxazepam—the individual’s platelet levels remained below the normal range. On May 3, 2022, she was hospitalized for additional diagnostic evaluations and therapeutic measures due to ecchymosis in her limbs and a decreased platelet count of 12 × 10^9^/L. At the time of admission, the patient had discrete petechiae on all four extremities, mainly on both lower limbs. The patient could respond simply and exhibited stable emotions. Laboratory tests indicated a white blood cell (WBC) count of 2.2 × 10^9^/L, a red blood cell (RBC) count of 2.58 × 10^12^/L, hemoglobin (HGB) levels at 90 g/L, a platelet (PLT) count of 7 × 10^9^/L, a procalcitonin (PCT) level of 0.01 ng/mL, and a C‐reactive protein (CRP) level of 24.80 mg/L. No evidence of platelet clumping was found in the peripheral blood smear. CT scans indicated chronic bronchial lesions and slight inflammation in the left lower lung. Considering the inconsistency with the infection course, antibiotics were not initiated. A prescription was made for the infusion of 14 units of type AB platelets and 75 mg of eltrombopag orally once daily. On June 5, laboratory tests showed WBC of 3.4 × 10^9^/L, HGB of 94 g/L, PLT of 11 × 10^9^/L, PTS of 10.1s, FIB of 4.42 g/L, and D2 of 2.26 mg/L and positive results for GPIIb/IIIa and GPIb/IX in platelet autoantibodies. On May 11, after platelet transfusion, the patient’s limb petechiae improved. A recheck of the blood routine showed WBC count of 1.9 × 10^9^/L, HGB level of 92 g/L, and PLT count of 31 × 10^9^/L. On May 15, the platelet count dropped sharply to 8 × 10^9^/L, along with oral bleeding, so the platelet transfusion therapy became necessary. On May 18, a routine hematological examination revealed WBC count of 1.8 × 10^9^/L, RBC count of 3.01 × 10^12^/L, HGB level of 102 g/L, and PLT count of 16 × 10^9^/L. Following this, a bone marrow aspiration was performed, which indicated moderate marrow hyperplasia, showing 15 megakaryocytes per slide. However, the subsequent biopsy revealed mild hyperplasia accompanied by a decrease in the number of megakaryocytes. The diagnosis considered ITP. Since glucocorticoids directly affect the central nervous system, using these drugs, especially in medium–high doses and for long term, significantly raises the risk of inducing or worsening psychotic symptoms in people prone to mental disorders like schizophrenia. On 25 May, the treatment regimen was modified to include cyclosporine 75 mg twice daily (equivalent to 3 mg/kg/day) and eltrombopag 50 mg once daily. To enhance the treatment effect, we increased diltiazem to 30 mg twice daily as it inhibits CYP3A4 to raise cyclosporine levels. On June 3, laboratory results indicated WBC count of 4.3 × 10^9^/L, RBC count of 3.35 × 10^12^/L, HGB level of 111 g/L, and PLT count of 36 × 10^9^/L. By June 10, there was an improvement in the platelet count, which increased to 53 × 10^9^/L. Follow‐up assessments conducted until November 11 indicated that the patient’s platelet count had risen to 115 × 10^9^/L (Figure 1), with cyclosporine A concentration ranging from 72.50–191.00 μg/L, liver and kidney functions within normal range, and no bleeding events occurred. The follow‐up period was extended for an additional three years, concluding in May 2025, during which no recurrence of ITP was noted.

**Figure FIGURE 1:**
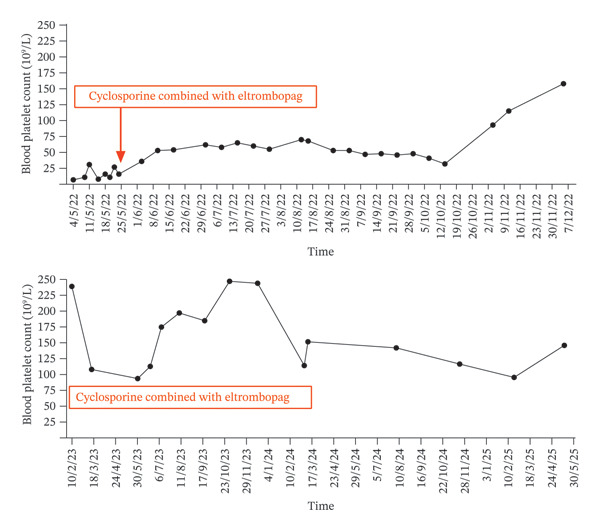
Platelet counts at the time of treatment and follow‐up.

## 3. Discussion

Oxaliplatin is a commonly utilized third‐generation platinum‐based chemotherapy agent that, when used in conjunction with fluoropyrimidine drugs, offers significant benefits to high‐risk Stage II and III colorectal cancer patients undergoing adjuvant chemotherapy. While clinicians possess a comprehensive grasp of prevalent adverse effects, including myelosuppression and peripheral neuropathy, oxaliplatin‐induced ITP is relatively rare and may be generally underrecognized. Currently, there exists a notable absence of evidence‐based guidelines to inform clinical decision‐making regarding this particular condition.

The mechanism of oxaliplatin‐induced ITP involves the development of antibodies against specific glycoprotein complexes on platelets, and repeated drug administration is a prerequisite for its occurrence. Hemorrhage is a common complication. Given that the patient’s condition can deteriorate rapidly, early diagnosis and treatment are extremely important. The most important aims of clinical therapy are raising platelet counts to safe levels to decrease the morbidity from bleeding and reduce mortality rates [2].

The following represents a patient with colon cancer who underwent adjuvant chemotherapy; after six cycles of the course, she presented with symptoms of bleeding and Grade IV thrombocytopenia. Thus, based on the clinical characteristics, his symptoms point toward chemotherapy‐associated acute thrombocytopenia, hence the initial diagnosis being bone marrow suppression. However, this patient’s platelet count failed to recover within the normal range after the period of bone marrow suppression, and bleeding symptoms repeatedly appeared. The laboratory tests showed a low platelet count, and the test for platelet autoantibody showed positivity to GPIIb/IIIa and GPIb/IX. Bone marrow aspiration revealed moderate megakaryocytic hyperplasia with maturation arrest. After ruling out other causes of thrombocytopenia, oxaliplatin‐induced ITP was diagnosed.

No evidence‐based guideline has been presented to guide clinicians on how to handle such cases, and the use of glucocorticoids is usually prescribed as treatment in the acute phase [3]. Optimal treatments other than glucocorticoids remain uncertain. Case reports also reflect this variability. For example, in two patients with acute immune‐mediated thrombocytopenia, platelet counts rapidly increased after discontinuing oxaliplatin [4]. In another case, the platelet count of a patient returned to above normal levels 9 days after receiving steroid therapy (intravenous methylprednisolone followed by oral prednisolone) [5]. A patient with advanced pancreatic cancer, despite receiving methylprednisolone (100 mg) and platelet transfusions, did not show an increase in platelet levels, but later an elevation above normal levels (over 150000/mL) was observed after plasma exchange and continued methylprednisolone treatment within a week [6]. And in one case, a patient who did not improve after four consecutive days of steroid monotherapy (dexamethasone 40 mg/day) showed a return of platelet counts to normal levels within 2 days after the addition of intravenous immunoglobulin [7].

Given that this patient’s disease course has exceeded 3 months, it is considered persistent, and active treatment is necessary to prevent chronicity and intractability. The patient’s history of psychiatric illness limits the use of steroids, and the monotherapy with eltrombopag has been ineffective. Because of the possibility of a chronic nature of the illness, a trial of a combination of cyclosporine and eltrombopag was tried. Cyclosporine, an immunosuppressive agent, is clinically used to treat ITP. Its bioavailability and plasma concentration can be increased by combining diltiazem. Combination regimens including cyclosporine have also been reported in meta‐analyses to markedly increase platelet counts [8]. Eltrombopag, an orally active nonpeptide thrombopoietin receptor agonist, was the first such agent to be licensed for the treatment of adult chronic ITP. It can stimulate the proliferation and differentiation of megakaryocytes in the bone marrow. A single‐center study of refractory ITP that did not respond to eltrombopag monotherapy demonstrated that a total of 76.2% (16/21) of the patients responded to the combination of low‐dose cyclosporine (3 mg/kg/d, with an initial target concentration of 75–120 ng/mL) and eltrombopag and observed complete response in 81.3% (13/16) of the patients [9]. During a median follow‐up of 180 days, there were no relapses, and 70% of patients successfully discontinued or reduced the concurrent ITP medications. A separate study demonstrated that the combination of eltrombopag and low‐dose rituximab effectively treated four patients suffering from refractory ITP [10]. Following the implementation of combination therapy using cyclosporine and eltrombopag, the patient’s platelet count rose above 50 × 10^9^/L within a 2‐week period. Remarkably, after 6 months of ongoing treatment, the platelet levels normalized, indicating a significant and positive response to the therapy. These findings would make a synergistic effect of cyclosporin and eltrombopag both on platelet production and/or destruction more plausible.

## 4. Conclusions

We here report a good therapeutic response in a psychiatrically comorbid patient with oxaliplatin‐induced ITP, despite drug contraindications and unsatisfactory response to eltrombopag administered alone. A very promising response rate may be achieved by a combination treatment of cyclosporine and eltrombopag, associated with a gradual normalization of platelet count. We hope that this outcome can assist other physicians in selecting appropriate treatment methods in similar situations.

## Author Contributions

All authors have contributed to this manuscript in terms of planning, conception and design, writing, and editing the final manuscript.

## Funding

This study was supported by a grant from Traditional Chinese Medical Science and Technology Plan of Zhejiang Province (2022ZB172) and Key Discipline Construction Project of Traditional Chinese Medicine in Zhejiang Province in 2024 (2024‐XK‐32).

## Ethics Statement

The authors have nothing to report.

## Consent

Written informed consent was obtained from the patient for publication of this case report and any accompanying images. A copy of the written consent is available for review by the Editor‐in‐Chief of this journal.

## Conflicts of Interest

The authors declare no conflicts of interest.

## Data Availability

All data generated or analyzed during this study are included in this published article.
